# Expression of Retinoic Acid Receptor (RAR) α Protein in the Synovial Membrane from Patients with Osteoarthritis and Rheumatoid Arthritis

**Published:** 2007-03

**Authors:** Zisakis A, Katsetos Cd, Vasiliou Ad, Karachalios T, Sakkas Li

**Affiliations:** 1 *Departments of Internal Medicine, Thessaly University School of Medicine, Larisa, Greece;*; 2 *Department of Pediatrics, Drexel University College of Medicine and Department of Pathology and Laboratory Medicine, St. Christopher’s Hospital for children, Philadelphia, PA, USA;*; 3 *Departments of Orthopedics, Thessaly University School of Medicine, Larisa, Greece*

**Keywords:** osteoarthritis, rheumatoid arthritis, retinoic acid receptor, synovial membrane

## Abstract

Retinoic acid receptors (RAR) are expressed in inflammatory cells and, through ligand binding, play an important role in cell proliferation and differentiation, as well as in regulation of cytokine and matrix metalloproteinase (MMP) production. Inflammatory cytokines and MMPs play a significant role in cartilage destruction in osteoarthritis (OA) and in joint destruction in rheumatoid arthritis (RA), the prototype of inflammatory arthritis. To determine if RARα is expressed in the synovial membrane (SM) of patients with OA and compare it with RA, SM biopsy samples were used in this study which were from 31 patients with late OA and 14 patients with late RA. Cryostat sections were studied by immunochemistry using a RARα-specific antibody. All SM samples from OA and RA patients exhibited cellular localization for RARα. Immunoreactivity was present in mononuclear inflammatory cells, endothelial cells, synovial lining cells, and fibroblasts. Inflammatory infiltrates were interstitial and nodular. Roughly one half of mononuclear cells in the inflammatory nodules in OA and RA were positive for RARα. The conclusion is that the presence of RARα in SM of patients with OA and RA suggests that RARs may play a role in the immunomodulation of synovial inflammation and therefore can be a potential target of therapeutic intervention in these arthritides.

## INTRODUCTION

Retinoid receptors are nuclear receptors for retinoic acid isoforms, all-trans retinoic acid and 9-cis retinoic acid, both of which are active derivatives of vitamin A. Two families of retinoid receptors have been recognized: retinoic acid receptors (RAR), activated by all-trans retinoic acid and 9-cis retinoic acid, and retinoic X receptors (RXR) activated by 9-cis retinoic acid. Each family has α, β and γ isotypes, each isotype comprising additional isoforms, which arise by alternative splicing and alternative promoter usage (1). Retinoids are multicellular immunomodulators, including humam and murine thymothyces, Langerhans’ cells, natural killer cells, and T lymphocytes (2). Both retinal and retinoic acid enhance the anti-CD3 monoclonal antibody-mediated activation and proliferation of human T cells (2). Also, retinoic acid induces RAR gene expression in murine T lymphocytes whilst retinoic acid and RAR function as ligand-inducible transcriptional enhancer factors in T cells (3). Upon binding to their cognate ligands, RARs regulate the expression of many genes involved in immune-mediated inflammation, including cytokines, metalloproteases (MMP), and maturation of dendritic cells (4-6). In particular, RARs activate the expression of some genes by interacting with their promoter regions, or inhibit the expression of other genes by binding to c-jun/c-fos and thus antagonizing AP-1 function (6). RARα inhibits, by antagonizing AP1, the gene expression of collagenase by fibroblasts and monocytes (5, 6), transforming growth factor (TGF)-β (7) and interleukin(IL)-6 (4).

Rheumatoid arthritis (RA) is the prototype of inflammatory joint disease that leads to joint destruction. RA synovial membrane (SM) exhibits mononuclear cell infiltrates, and synovial cell proliferation. The cartilage and bone destruction in RA are largely mediated by proinflammatory cytokines and matrix metalloproteases (MMP) (8). Osteoarthritis (OA) exhibits varying degrees of mononuclear cell infiltration of the SM, and articular cartilage destruction. In OA, cartilage destruction is mediated by pro-inflammatory cytokines and MMPs (9).

We have hypothesized that the immune-mediated inflammatory responses in OA and RA can be modulated by retinoid receptors (RARs/RXRs) and their ligands. To this end, we have specifically investigated the expression and cellular distribution of RARα protein in inflammatory infiltrates affecting the SM of patients with OA and RA.

## METHODS

### Patients

Thirty one patients with OA [5 men, 26 women; mean age 68.3 ± 7.0 (SD)] and 14 patients with RA [3 men, 11 women; age, mean 65.0 ± 7.9 (SD)] were included in the study. Incidental SM specimens were obtained during joint replacement surgery and kept in OCT at -80°C.

### Antibody

A rabbit polyclonal anti-RARα antibody was used (C-20, Santa Cruz Biotechnology, and Santa Cruz, CA). C-20 is an affinity purified, peptide-specific polyclonal antibody directed against the amino acid sequence 443-462 of the C-terminus of RARα1 which is identical to the corresponding region of RARα2. C-20 does not cross-react with RARβ or RARγ isotypes, as previously demonstrated by Western blot analysis (10). An anti-CD3 (mouse anti-human IgG1, clone UCHT1, R&D systems, Mineapolis, MN) and anti-CD68 (mouse anti-human IgG1, clone KP1, Dako, Glostrup, Denmark) monoclonal antibodies, markers of T cells and macrophages, respectively, were used in representative biopsy samples.

### Immunohistochemistry

Cryostat sections (6 μm) were air dried for 1 hour and then fixed in cold acetone at -20°C for 1 hour. Then, sections were incubated with H_2_O_2_ 0.3% solution. Staining for RARα was carried out with the avidin–biotin complex immunoperoxidase method using the rabbit IgG ABC Elite Vectastain kit (Vector Laboratories, Burlingame, CA) and the c-20 polyclonal antibody (8). Sections were incubated with goat (ABC rabbit IgG kit) serum for 20 min to reduce non specific binding followed by incubation for 1 hour with anti-RARα antibody (dilution 1:200) at room temperature. Then sections were incubated with anti-rabbit biotynylated avidin horse-raddish peroxidase complex and developed with the Vector DAB. As control for the antibody, a rabbit IgG was used (Sigma Chemicals Co., Saint Louis MO). Finally, sections were counterstained with Papanicolaou Haematoxylin.

## RESULTS

**Figure 1:**
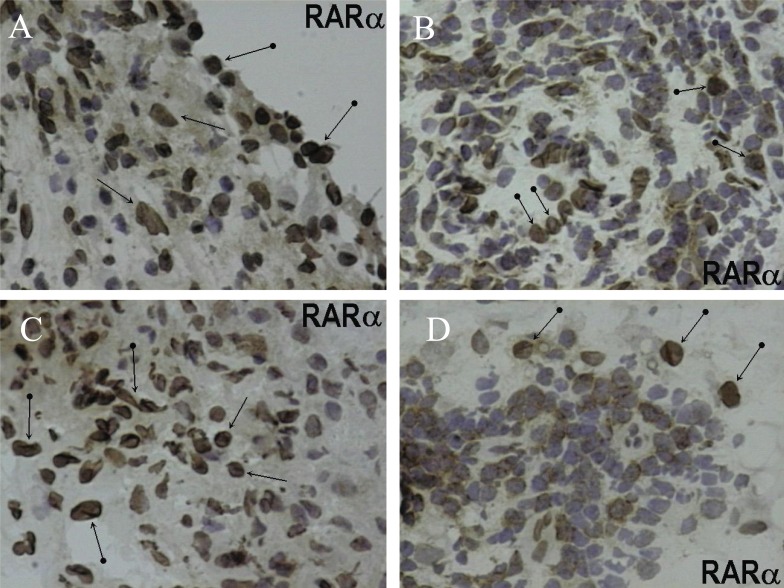
Avidin-biotin complex peroxidase method with light hematoxylin counterstaining of synovial membrane. The upper two panels (A, B) are sections from patients with OA and the lower two panels (C, D) are from patients with RA. A depicts prominent nuclear RARα staining in synoviocytes (clubbed arrows), and interstitial mononuclear cells with round nuclei. B shows a nodular inflammatory infiltrate exhibiting RARα staining in mononuclear round cells consistent with lymphocytes (clubbed arrows); the majority of these cells are CD3+ T cells but a subpopulation of these infiltrates corresponds to CD68+ monocytes /macrophages in immediately adjacent tissue sections (not shown). C depicts nuclear RARα staining in interstitial mononuclear cell infiltrates (thin arrows) and putative fibroblasts (cells with fusiform nuclei-clubbed arrows). D shows a nodular inflammatory infiltrate exhibiting RARα staining in mononuclear round cells consistent with lymphocytes; robust RARα localization is demonstrated in occasional cells with larger, oval nuclei consistent with monocytes/macrophages (clubbed arrows). (Original magnifications X400).

All specimens from patients with OA and RA exhibited immunoreactivity for RARα. Immunohistochemical localization was detected in the nuclei of synovial lining cells, fibroblasts, endothelial cells, and in subpopulations of mononuclear inflammatory cells exhibiting an interstitial and/or nodular distribution with a perivascular (angiocentric) predilection. The latter were comprised predominantly of T cells and-to lesser extent-cells of the monocyte-macrophage lineage, as determined by CD3 and CD68 localizations, respectively. The cellular distribution and intensity of RARα staining was identical in SM from OA and RA. In inflammatory nodules, between 30% and 40% of mononuclear inflammatory cells were immunoreactive for RARα (Figure 1).

## DISCUSSION

The present study demonstrated RARα protein expression in SM from patients with OA and RA. Previous studies demonstrated mRNA expression of RAR iso-types in various cells. Peripheral blood B cells constitutively express RARα and RARγ mRNA and very little RARβ mRNA (11) and T cells from adenoid tissues (12) constitutively express RARα and RARγ mRNA and very little if any RARβ mRNA. In adenoid T and B cells, the RARα1 isoform is constitutively expressed whereas the RARα2 isoform is induced by all-trans retinoic acid (12). We have previously demonstrated localization of RARα in tumor infiltrating mononuclear cells in fibroblasts and in vascular endothelial cells from ovarian cancer specimens (10).

The three RAR isotypes are functionally distinct and regulate the expression of different genes in immune cells. RARα mediates proliferation of normal T cells by increasing IL-2 secretion (13). In addition, it inhibits activation-induced apoptosis and Fas ligand (FasL) expression of CD4 T cells (14). It may be reminded that apoptosis plays a major role in shaping the T cell repertoire, and to this end retinoic acid can have multiple different effects on apoptosis. For instance, retinoic acid can induce apoptosis, enhance dexamethasone-induced death and inhibit activation-induced death of T cells (15). Apoptosis of T cells is mediated by retinoid ligands through stimulation of RARγ, whilst, on the other hand, specific stimulation of RARα prevents both RARγ-dependent and T cell receptor-mediated cell death (15, 16). In lymphocytes, RARα regulates the expression of CD38, a transmembrane protein functioning as adhesion and signal transduction molecule (17). In dendritic cells, via interaction with TNFα, RARα –dependent pathways increase MHC class II and co-stimulatory molecule expression, and enhance antigenspecific T cell responses (18). In contrast, in the absence of inflammatory cytokines, RARs mediate dendritic cell apoptosis (18).

The high expression of RAR in infiltrating mononuclear cells suggests that the PAR pathway may be involved in the modulation of inflammation and joint destruction in both OA and RA. Retinoids have been used in the treatment of RA with poor efficacy and serious side effects. In animal models of arthritis, a RAR antagonist blocked the clinical progression of arthritis with concomitant reduction of MMP expression by fibroblasts (19). On the other hand, a RARa selective agonist has been shown to act as an immunosuppressant by inhibiting cytotoxic T cells and alloantigen-stimulated production of pro-inflammatory cytokines in cardiac transplantation (20). This underscores the complexity underlining the action of RAR selective agonists and antagonists in inflammatory processes and renews interest in the therapeutic role of retinoids in the management of RA and certain forms of OA.
